# Analysis of long non-coding RNA and mRNA expression in bovine macrophages brings up novel aspects of *Mycobacterium avium* subspecies *paratuberculosis* infections

**DOI:** 10.1038/s41598-018-38141-x

**Published:** 2019-02-07

**Authors:** Pooja Gupta, Sarah Peter, Markus Jung, Astrid Lewin, Georg Hemmrich-Stanisak, Andre Franke, Max von Kleist, Christof Schütte, Ralf Einspanier, Soroush Sharbati, Jennifer zur Bruegge

**Affiliations:** 1 Department of Mathematics and Informatics, Freie Universität Berlin, Berlin, Germany; 2 Institute for the Reproduction of Farm Animals Schönow Inc, Bernau, Germany; 3 Robert Koch-Institute, Department Infectious Diseases, Berlin, Germany; 4 Institute of Clinical Molecular Biology, Christian-Albrechts-University Kiel, Kiel, Germany; 5 Institute of Veterinary Biochemistry, Department of Veterinary Medicine, Freie Universität Berlin, Berlin, Germany; 6 Department of Mathematics for Life and Materials Sciences, Zuse Institute Berlin, Berlin, Germany

## Abstract

Paratuberculosis is a major disease in cattle that severely affects animal welfare and causes huge economic losses worldwide. Development of alternative diagnostic methods is of urgent need to control the disease. Recent studies suggest that long non-coding RNAs (lncRNAs) play a crucial role in regulating immune function and may confer valuable information about the disease. However, their role has not yet been investigated in cattle with respect to infection towards Paratuberculosis. Therefore, we investigated the alteration in genomic expression profiles of mRNA and lncRNA in bovine macrophages in response to Paratuberculosis infection using RNA-Seq. We identified 397 potentially novel lncRNA candidates in macrophages of which 38 were differentially regulated by the infection. A total of 820 coding genes were also significantly altered by the infection. Co-expression analysis of lncRNAs and their neighbouring coding genes suggest regulatory functions of lncRNAs in pathways related to immune response. For example, this included protein coding genes such as *TNIP3*, *TNFAIP3* and *NF-κB*2 that play a role in NF-κB2 signalling, a pathway associated with immune response. This study advances our understanding of lncRNA roles during Paratuberculosis infection.

## Introduction

*Mycobacterium avium* subspecies *paratuberculosis* (MAP) is the causative agent of Paratuberculosis or Johne’s disease, a chronic enteritis in ruminants. Animals suffer from prolonged diarrhoea and progressive wasting^1^. The disease causes major economic losses for the dairy industry worldwide. A causal role of MAP in the pathogenesis of human Crohn’s disease is discussed controversially^2^.

It is estimated that around 31–71% of cattle herds in European countries are infected^3–5^. Due to the unique pathogenesis of MAP, diagnosis of Paratuberculosis is difficult, especially in the early phase of infection. Neonates and calves in their first few months of life are most susceptible to MAP^6^. The pathogen is faecal-orally transmitted via ingestion of contaminated colostrum, water or feed. Intestinal epithelial cells are the main port of entry for MAP, which preferentially invades enterocytes and specialized epithelial cells known as M cells^1^. These cells then translocate MAP from the intestinal lumen to the submucosa where MAP is taken up by subepithelial macrophages^1^. The interaction of MAP and macrophages determines whether the host is able to eliminate the pathogen or if infection is being established. Control of MAP infections depends on several factors including an early protective Th1 cell response promoting INF-γ release and activation of antimicrobial mechanisms in macrophages^7^. However, like many other Mycobacteria, MAP is able to modulate macrophage response to enhance its intracellular persistence and survival. For example, it inhibits phago-lysosomal maturation in macrophages in order to avoid acidification and bacterial killing, and it also impairs antigen presentation to T cells^8^. Furthermore, MAP modulates INF-γ signalling or induces increased secretion of IL-10 to promote bacterial persistence and establish infection^9^.

Depending on the infection dose and animal health status, the incubation period for Paratuberculosis is long (>2 years). Around 10–15% of infected cattle develop clinical signs^6^. During the incubation time, animals show little or no clinical signs of infection^1^. Nevertheless, infected animals in the subclinical phase may shed MAP and serve as a direct source of transmission. Detection and management of such animals is crucial for the success of Paratuberculosis control measures. However, serum antibodies are usually not detectable in the preclinical phase. Faecal culture is considered as the “gold standard” for diagnosis but sensitivity in early subclinical infections is low and cultivation is protracted. Detection of MAP in faeces via PCR may be an alternative to faecal culture^10^, however, detection rates depend on the shedding status of the animal. Thus, the development of alternative diagnostic tools is of urgent need to control the disease.

The use of non-coding RNAs as a novel and promising diagnostic approach of infectious and non-infectious diseases has recently become a major focus of investigation^11,12^. It was shown that bacteria interfere with the expression of mammalian regulatory RNAs to modify immune signalling, autophagy, or the apoptotic machinery. Recently, a new class of regulatory RNAs, long non-coding RNAs (lncRNAs), was reported to play a crucial role in the regulation of eukaryotic gene expression. A rising amount of literature reports on specific involvement of lncRNAs in the host cell response towards bacterial infections^13^. Based on their size (larger than 200 nt) lncRNAs are distinguished from other non-coding RNAs such as microRNAs (miRNAs). They function as protein scaffolds, activators or inhibitors of transcription, antisense RNA or miRNA sponges^14^. They are known to possess low evolutionary conservation and exhibit lower levels of cellular concentration than protein coding genes but have a higher degree of tissue specificity^15^. Nonetheless, despite having a key role in the regulation of gene expression, lncRNAs remain poorly identified and annotated in domesticated animals in comparison to other species such as humans and mice^16^.

The objective of this study is to deepen the knowledge about the crucial interplay between bovine macrophages and MAP, and furthermore provide novel insight into the regulatory function of lncRNAs in cattle during MAP infection. To this end, we investigated the changes in mRNA expression profiles and the presence of potentially novel lncRNA candidates and their role in bovine macrophages with respect to MAP infection via RNA sequencing.

## Results

### Identification of putative lncRNA transcripts

**Figure 1:**
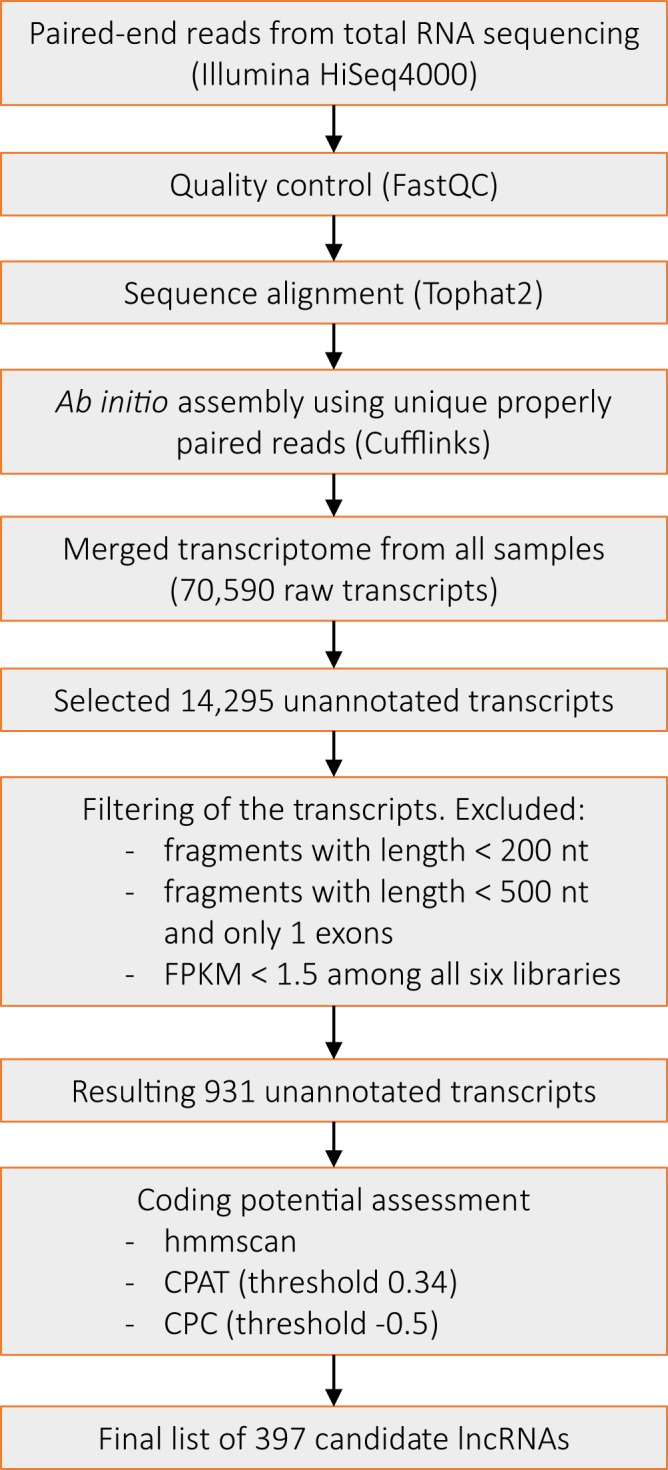
Pipeline for selecting lncRNA. A schematic overview of the pipeline employed for selecting candidate lncRNA based on relevant filtering criteria.

We analysed high-throughput RNA-Seq data derived from MAP infected and uninfected primary bovine macrophages. The alignment of paired-end reads resulted in concordant mapping rates of >90% for all libraries (Supplementary Table S1). We obtained 70,590 transcripts (Fig. 1) by assembling 240 million uniquely mapped and properly paired reads (35.9 to 43.0 million paired-end reads per sample). A computational pipeline (Fig. 1) adapted from earlier studies^17–21^ was implemented to characterize and identify candidate lncRNAs. Initially, 14,295 transcripts with the class codes ‘u’ and ‘x’ that have unknown annotations were retained to nominate putative lncRNAs. We obtained 931 unannotated transcripts (Fig. 1, Supplementary Table S2) after filtering out low quality assemblies such as those with a small fragment size (<200 bp; <500 bp for single-exon transcripts^20^) and low expression (FPKM <1.5 among all six libraries^17,20^). The selected lncRNAs were searched for known protein domains against the Pfam database^22^. In addition, their protein coding ability was assessed using coding potential calculator^23^ (CPC) and coding-potential assessment tool^24^ (CPAT). We included only those lncRNAs that showed no matching domain in the Pfam database, resulting in 490 candidates. Using a threshold cut-off score of −0.5 for CPC^18^, we obtained 425 non-coding candidate transcripts. Furthermore, CPAT provided 439 non-coding candidate transcripts with a cut-off threshold of 0.34. An intersection of all three approaches (Supplementary Fig. S1) provided a final list of 397 candidate lncRNAs (Supplementary Data 1a,b).

### Genomic characteristics of lncRNAs in cattle

**Figure 2:**
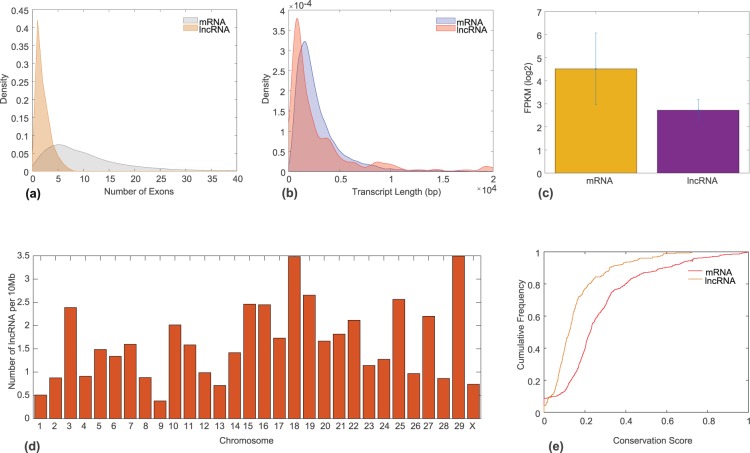
Genomic properties of the candidate lncRNA. (**a**) Density plot for the number of exons in mRNA and candidate lncRNA. (**b**) Density plot for the length of mRNA and candidate lncRNA. (**c**) Expression levels (FPKM) of mRNA and lncRNA. (**d**) Distribution of the candidate lncRNA across the cow genome. (**e**) Cumulative density plot for the conservation score of mRNA and candidate lncRNA.

LncRNAs have been shown to possess distinguishing features in comparison to other RNAs, e.g. lower levels of cellular concentration, fewer exons and shorter length than protein coding genes^16^. Therefore, we analysed the differences in number of exon, length, expression and genomic distribution between lncRNAs and 12,092 mRNAs expressed in our samples (Fig. 2). We observed that lncRNAs have fewer number of exon (median = 2) than mRNAs (median = 8; *P* = 4.12e-178, two-tailed Mann-Whitney U test, Fig. 2a). On an average, we found around 71% transcripts with less than three exons and almost 48% transcripts having single exon. We also found that the most frequently observed length of lncRNAs (median = 1,505) was smaller (Fig. 2b) than the length of mRNAs (median = 2,144; *P* = 8.18e-09, two-tailed Mann-Whitney U test). Furthermore, the gene expression levels differed significantly between candidate lncRNAs and mRNAs (Fig. 2c). The expression of lncRNAs (mean FPKM = 6.59) is lower compared with mRNAs (mean FPKM = 22.84; *P* = 2.37e-07, two-tailed Student’s t-test). Figure 2d shows the distribution of candidate lncRNAs across the genome of the cow. The average number of candidate lncRNAs per chromosome per 10 Mb was approximately 1.6. The distribution of candidate lncRNAs across the genome exhibited a pattern similar to the distribution of protein-coding genes expressed in our samples (Supplementary Fig. S2). Similar to mRNA distribution, chromosomes 18, 19, 25 and 29 contained more lncRNAs in comparison to the rest of chromosomes, with each containing >5% of candidate lncRNAs per chromosome per 10 Mb (Fig. 2d, Supplementary Table S3).

As it has been shown that lncRNAs are evolutionarily less conserved than protein-coding genes^15^, we investigated the conservation scores of our candidate lncRNAs. Using UCSC’s ‘liftover’ based conversion of genomic coordinates from the bosTau8 to the bosTau4 assembly, we retrieved the PhastCons scores^25^ for 282 candidate lncRNAs; the coordinates for remaining lncRNAs did not exist in the bosTau4 assembly. Figure 2e shows the cumulative density plot of the PhastCons scores for the lncRNAs and 500 randomly selected mRNAs (annotated in Ensemble GTF). As expected, we found that lncRNAs are not as conserved as protein-coding transcripts (Fig. 2e, *P* = 2.18e-11, two-tailed Student’s t-test). However, despite an overall low conservation score, we observed local regions with reasonable conservation across the transcripts (Supplementary Data 2).

### Differential expression of lncRNA and mRNA

**Figure 3:**
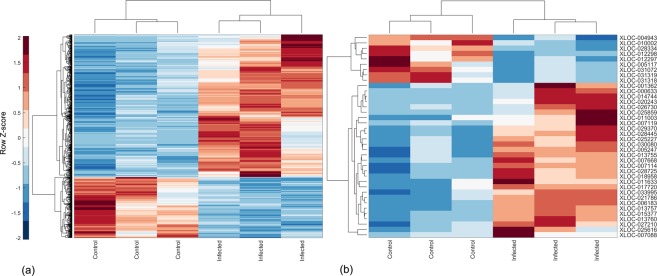
Cluster analysis of the differentially expressed genes. (**a**) A hierarchical heatmap showing differentially expressed mRNA in control and infected samples. (**b**) A hierarchical heatmap showing differentially expressed lncRNA candidates in control and infected samples. Red shows higher expression and blue shows lower expression.

In total, 820 protein-coding genes (Fig. 3a, Table 1, Supplementary Data 3, Supplementary Figs S3 and S4) and 38 lncRNAs genes (Fig. 3b, Table 2, Supplementary Data 4) were significantly differentially expressed (DE; corrected p-value for multiple testing using Benjamini-Hochberg technique^26^ p.BH <0.05). Among them, 347 upregulated and 156 downregulated genes as well as 25 upregulated and 7 downregulated lncRNAs showed distinct dysregulation with a log2 fold-change (L2FC) ≥ 1 or ≤ −1. For 20 out of these 38 DE lncRNAs, the closest neighbouring mRNAs were also significantly DE (11 upregulated and 1 downregulated for L2FC ≥ 1 or ≤ −1 and p.BH <0.05, Table 3).

**Table 1:** Top 20 upregulated and downregulated mRNAs.

Gene symbol/Ensemble ID	Chromosome	Base mean	L2FC*	p.BH^#^
*CCL8*	19	11904.20	6.53	3.02E-05
*CSF2*	7	2046.87	5.85	2.75E-03
*IFIT2*	26	2211.50	5.18	1.68E-04
*IL9*	7	28.77	4.97	1.12E-06
*LIF*	17	62.70	4.90	6.00E-04
*RSAD2*	11	35769.02	4.87	3.48E-10
*IFIT1*	26	34648.49	4.51	5.04E-04
*CSF3*	19	2155.09	4.44	2.06E-03
*PTX3*	1	1027.85	4.37	1.62E-02
*IL6*	8	12.19	4.32	8.55E-04
*TSG-6*	2	187.69	4.31	1.39E-02
*TACSTD2*	3	9.59	4.22	2.44E-02
*SLFN11*	19	71.56	4.21	4.89E-06
*TNF*	23	5526.87	4.21	1.41E-12
*ISG20*	21	255.51	3.99	1.98E-07
*CXCL2*	6	34701.65	3.87	2.29E-02
*CMPK2*	11	5036.52	3.82	3.63E-06
*INHBA*	4	640.91	3.80	1.52E-02
*MEFV*	25	2935.40	3.74	6.77E-08
*IL1B*	11	52671.40	3.73	1.45E-02
*ENSBTAG00000031795*	1	36.15	−3.87	1.56E-02
*GAS1*	8	39.31	−3.47	1.75E-04
*RAB42*	2	71.41	−3.42	5.73E-04
*PIK3IP1*	17	745.19	−3.40	9.17E-08
*SRMS*	13	35.83	−2.93	2.59E-04
*LPAR5*	5	35.97	−2.78	2.54E-02
*RARG*	5	93.86	−2.78	3.48E-10
*C19orf35*	7	93.20	−2.74	1.41E-02
*SGSM1*	17	157.99	−2.69	2.23E-09
*SMTNL2*	19	40.68	−2.69	1.75E-02
*TFAP4*	25	58.19	−2.60	6.61E-03
*SMAD6*	10	83.82	−2.54	8.08E-06
*CRACR2B*	29	30.58	−2.52	2.16E-02
*CBFA2T3*	18	177.92	−2.49	7.71E-05
*TRERF1*	23	164.64	−2.42	3.76E-04
*DBP*	18	174.75	−2.35	7.00E-06
*MEF2C*	7	66.10	−2.32	6.92E-03
*CBX8*	19	134.95	−2.27	2.14E-04
*NRADD*	22	310.52	−2.27	7.48E-07
*ENSBTAG00000046828*	21	33.24	−2.24	6.64E-03

*L2FC denotes the log2 fold-change. ^#^p.BH denotes the p-value corrected for multiple testing using Benjamini-Hochberg method.

**Table 2:** Top 10 upregulated and top 9 downregulated lncRNAs.

Gene ID	Chromosome	Base mean	L2FC*	p.BH^#^
*XLOC_000633*	1	613.81	4.68	1.57E-12
*XLOC_014744*	19	86.99	3.27	3.80E-07
*XLOC_025859*	3	37.60	3.15	6.31E-05
*XLOC_007088*	14	94.29	2.72	1.45E-02
*XLOC_021786*	26	63.96	1.96	2.26E-03
*XLOC_026730*	4	472.66	1.89	4.70E-03
*XLOC_007668* ^§^	14	309.43	1.76	4.86E-03
*XLOC_033995*	9	604.78	1.70	1.90E-02
*XLOC_001362*	1	2492.84	1.64	2.00E-02
*XLOC_018958*	23	265.57	1.56	1.24E-04
*XLOC_005117*	12	607.08	−2.24	2.26E-02
*XLOC_012297*	18	42.87	−1.83	8.13E-03
*XLOC_012298*	18	34.84	−1.77	7.81E-03
*XLOC_031319*	7	239.91	−1.60	1.20E-02
*XLOC_031318*	7	141.73	−1.56	1.52E-02
*XLOC_004943*	12	574.63	−1.35	1.40E-03
*XLOC_028334*	5	1178.68	−1.02	4.24E-05
*XLOC_010002*	16	399.07	−0.85	4.00E-02
*XLOC_031072*	7	486.79	−0.81	4.43E-02

*L2FC denotes the log2 fold-change. ^#^p.BH denotes the p-value corrected for multiple testing using Benjamini-Hochberg method. ^§^The region XLOC_007668 assembled by Cuffmerge contained both lncRNA and mRNA DCSTAMP loci. Therefore, here for we provide L2FC and p.BH estimates for region encompassed by the lncRNA transcripts within the XLOC_007668 region.

**Table 3:** List of differentially expressed mRNA neighbours to differentially expressed lncRNAs.

Gene symbol	L2FC*	p.BH^#^	lncRNA neighbour	Distance (bp)	L2FC*	p.BH^#^
*MAF*	−1.89	2.26E-03	*XLOC_012297*	14863	−1.83	8.13E-03
			*XLOC_012298*	14231	−1.77	7.81E-03
*TNRC6B*	−0.55	2.11E-02	*XLOC_028334*	3506	−1.02	4.24E-05
*ZMIZ2*	0.78	3.09E-02	*XLOC_027210*	4650	1.27	2.11E-02
*CSF2RA*	0.78	2.40E-03	*XLOC_025227*	23467	1.04	6.46E-03
*RHBDF2*	0.86	5.02E-05	*XLOC_013760*	9945	0.94	4.54E-03
			*XLOC_013755*	14339	1.04	1.20E-02
			*XLOC_013757*	12713	1.08	9.79E-05
*RASSF3*	0.94	2.56E-02	*XLOC_028725*	2463	1.04	7.35E-03
*RELL1*	1.18	1.09E-02	*XLOC_030080*	14289	1.43	4.41E-04
*IL17REL*	1.30	3.02E-05	*XLOC_029370*	791	1.27	6.70E-05
*TNIP3*	1.42	1.68E-02	*XLOC_011003*	5602	1.27	3.64E-02
*NFKB2*	1.50	7.94E-05	*XLOC_021786*	325	1.96	2.26E-03
*PIM1*	1.55	2.35E-05	*XLOC_018958*	515	1.56	1.24E-04
*DCSTAMP*	1.57	1.72E-02	*XLOC_007668*	6809	1.76	4.86E-03
*TNFAIP3*	1.76	5.25E-03	*XLOC_033995*	1049	1.70	1.90E-02
*RNF213*	1.91	1.41E-02	*XLOC_014744*	24052	3.27	3.80E-07
*MX2*	2.25	2.56E-02	*XLOC_001362*	96	1.64	2.00E-02
*IFI44L*	2.57	3.72E-05	*XLOC_025859*	2065	3.15	6.31E-05
*PIM3*	2.63	7.62E-09	*XLOC_028445*	2456	1.18	2.72E-04

*L2FC denotes the log2 fold-change. ^#^p.BH denotes the p-value corrected for multiple testing using Benjamini-Hochberg method.

### Analysis of neighbouring protein-coding genes

**Figure 4:**
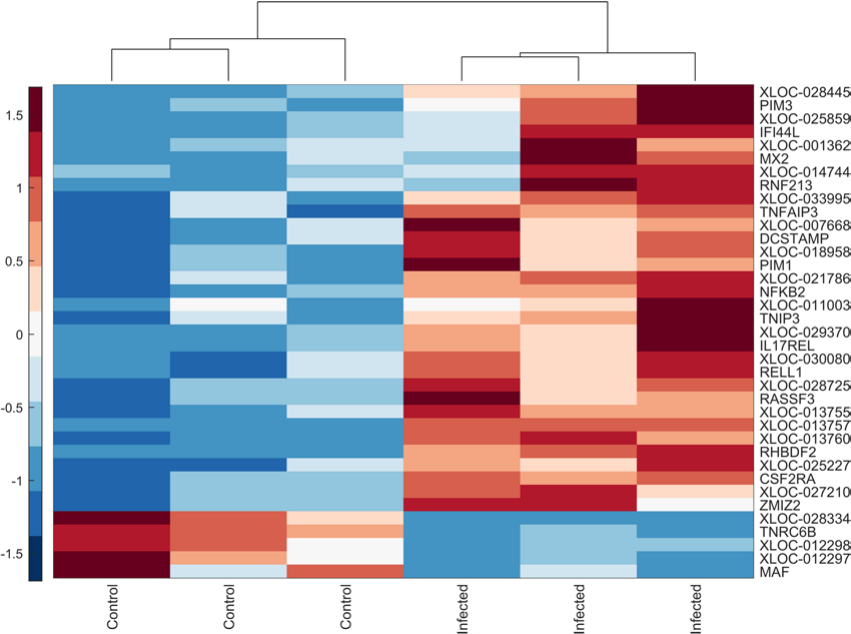
Co-expression analysis. A hierarchical heatmap showing the co-expression of differentially expressed lncRNA candidates and its corresponding differentially expressed neighbour mRNA. Red shows higher expression and blue shows lower expression.

The average distance between lncRNAs and their closest neighbour was 34 kb (Supplementary Data 5). DE neighbouring mRNAs were present within shorter distances (mean = 7.3 kb, Table 3). We also analysed the expression levels of DE neighbours. Interestingly, a high positive correlation (Pearson correlation r >0.80, *P* < 0.05 (two-tailed Student’s t-test); performed in MATLAB (version R2017b) using the function *corr*) was observed between the pair of DE lncRNA and their corresponding DE neighbouring protein-coding gene (except the pair *XLOC_012298-MAF*; Fig. 4). The pair exhibited a congruent co-expression, i.e. the change in expression was in the same direction. As shown in Fig. 4, *TNRC6B* and *MAF* were downregulated similar to their corresponding non-coding RNA neighbours (Table 3). On the other hand, for the upregulated lncRNAs, their corresponding neighbouring genes *ZMIZ2*, *TNIP3*, *TNFAIP3*, *RNF213*, *RHBDF2*, *RELL1*, *RASSF3*, *PIM3*, *PIM1*, *NFKB2*, *MX2*, *IL17REL*, *IFI44L*, *DCSTAMP* and *CSF2RA* were also upregulated (Fig. 4, Table 3).

### Enrichment analysis

**Figure 5:**
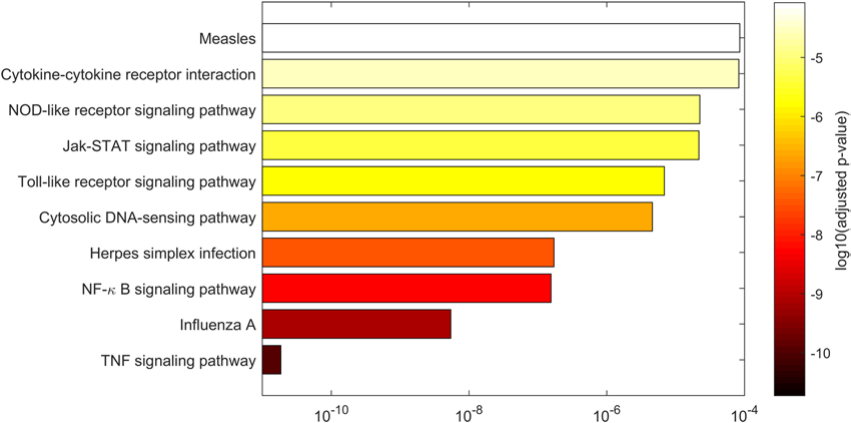
Top ten KEGG terms for the differentially expressed mRNA. An overview of the significantly enriched KEGG terms (p.BH <0.05) for the differentially expressed mRNA in the form of a bar plot. The length of the bar plot indicates p.BH i.e. the adjusted p-value obtained using Benjamini-Hochberg method for multiple test correction.

The enrichment analysis of 820 DE mRNAs pointed out 26 significantly enriched KEGG pathways (EASE score^27^ <0.05, p.BH <0.05) with ‘TNF signalling pathway’, ‘Influenza A’, ‘NF-κB signalling pathway’, ‘Herpes simplex infection’, ‘Cytosolic DNA-sensing pathway’, ‘Toll-like receptor signalling pathway’, ‘Jak-STAT signalling pathway’, ‘NOD-like receptor signalling pathway’, ‘Cytokine-cytokine receptor interaction’ and ‘Measles’ being the ten most enriched pathways (Fig. 5). Similarly, the significant gene ontology terms (EASE score <0.05, p.BH <0.05) associated with biological processes included ‘cellular response to lipopolysaccharide’, ‘negative regulation of viral genome replication’, ‘positive regulation of NF-κB transcription factor activity’, ‘innate immune response’, ‘defence response to virus’, ‘inflammatory response’, ‘immune response’, ‘positive regulation of interleukin-6 production’, ‘lipopolysaccharide-mediated signalling pathway’ and ‘regulation of transcription from RNA polymerase II promoter’ (Supplementary Fig. S5). Significantly enriched UniProtKB keywords (EASE score <0.05, p.BH <0.05) included ‘Cytokine’, ‘Activator’, ‘Transcription regulation’, ‘Innate immunity’, ‘Inflammatory response’ and ‘Transcription’ (Supplementary Fig. S5). We also performed an enrichment analysis of the neighbouring mRNA genes of DE lncRNAs (Supplementary Fig. S6). Pathways and functions associated with immune response such as NF-κB signalling (*TNFRSF11A*, *NFKB2*, *TNFAIP3*), organelle fission (*MX1*, *MX2*) and cytokine production (*MAF*, *NFAT5*) were enriched (EASE score <0.05).

### Overlap with previously published lncRNAs in cattle

In total, we found 172 of 397 candidate lncRNA transcripts in our dataset to have an overlap (i.e. the number of nucleotides shared between the two overlapping features on the same strand) with previously published transcripts (Supplementary Data 6) in cattle from five different sources mentioned below. 225 of our candidate transcripts were unique, having no match to any transcript from the sources investigated. We identified 85 and 63 transcripts having a match with a non-coding RNA in the NONCODE (NONCODE2016_bosTau6) and ALDB (ALDB.cow.lincRNAs.v1.0) databases (Supplementary Table S4). 36, 53 and 44 transcripts found a match with a non-coding RNA listed in the publications from Weikard *et al*.^18^, Billerey *et al*.^19^ and Koufariotis *et al*.^28^, respectively (Supplementary Table S4). No intersection was found among the five sources (Supplementary Fig. S7). 80 transcripts had an overlap of more than 90% with a previously published non-coding RNA (Supplementary Data 6, Sheet 1, Column 20).

### RT-qPCR analysis reflects expression of selected lncRNAs determined by RNA sequencing

**Figure 6:**
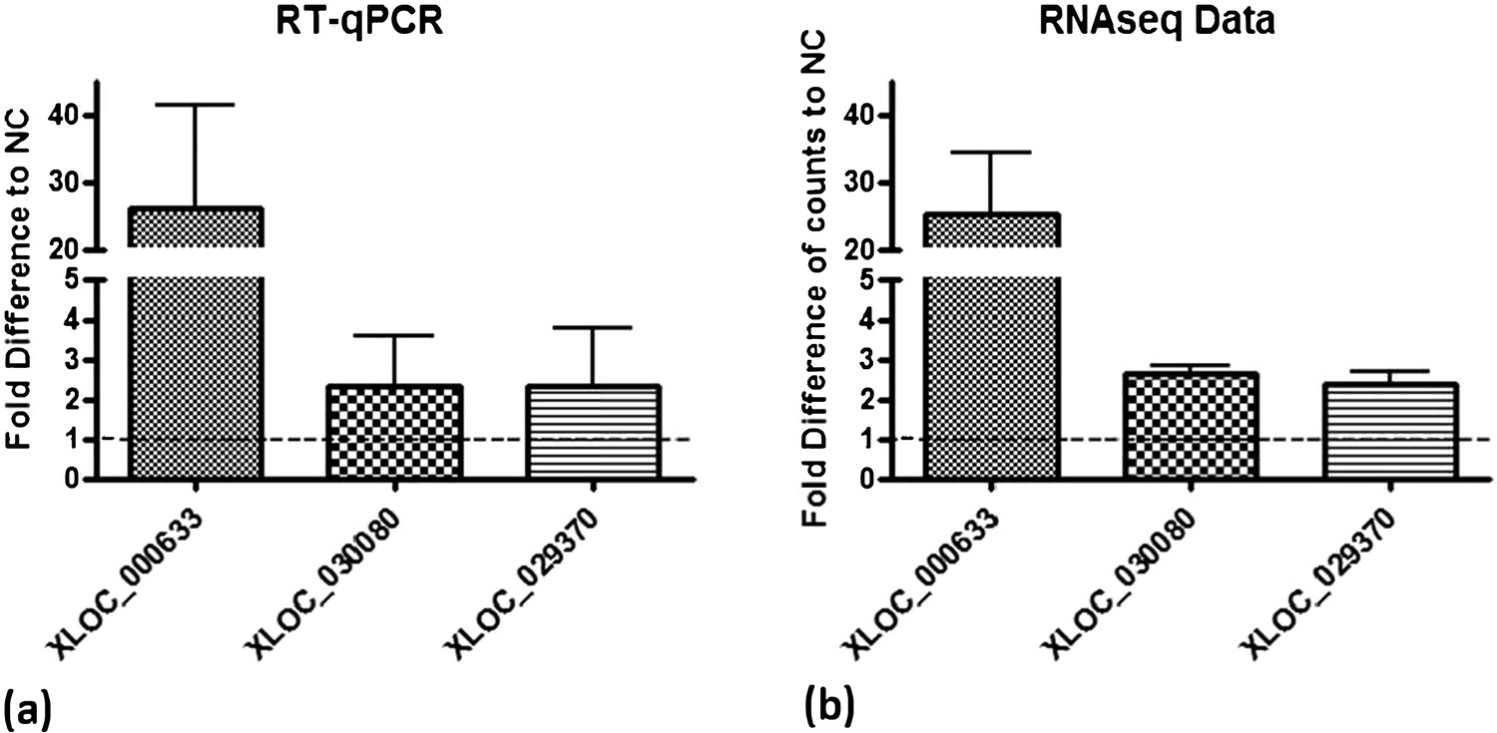
RT-qPCR validation. (**a**) Expression of selected lncRNAs validated with RT-qPCR and (**b**) in comparison fold differences of transcript counts to negative control (NC) determined by RNA sequencing.

To validate the RNA sequencing data and the implemented lncRNA nomination pipeline, expression of three randomly selected lncRNAs was analysed by means of RT-qPCR. Expression patterns determined by RT-qPCR reflects the RNA sequencing data (Fig. 6a,b). *XLOC_000633* was highly upregulated in infected samples compared to the controls (analysis with RT-qPCR resulted in a mean fold difference = 26.09, the mean fold difference calculated for sequencing counts compared to negative controls = 25.24). *XLOC_030080* and *XLOC_029370* were moderately upregulated by the infection compared to the controls. RT-qPCR resulted in a mean fold difference = 2.32 for *XLOC_030080* and a mean fold difference = 2.34 for *XLOC_029370*. The mean fold difference calculated for sequencing counts was 2.64 for *XLOC_03008* and 2.38 for *XLOC_029370*.

## Discussion

Paratuberculosis is being recognized as a major problem affecting animal health, farming and the dairy industry worldwide. Because of specific virulence mechanisms employed by the pathogen, infection may result in a long-term subclinical carriage and may finally lead to diarrhoea and progressive wasting. In this study, we used RNA-Seq data to investigate the alteration in genomic expression profiles of mRNA and lncRNA in bovine macrophages upon MAP infection. A comprehensive computational pipeline adapted from earlier studies^17–21^ was implemented for the identification of lncRNAs in cattle. We inferred the putative biological roles of the identified candidate lncRNAs by assessing the function of neighbouring protein-coding genes and their co-expression patterns. The current study brings up novel information about the early immune response of bovine macrophages upon MAP infection and points out the importance of regulatory RNAs.

### Analysis of mRNAs confirms and broadens existing knowledge on early immune response of macrophages upon MAP infection

One aim of the present study was the analysis of mRNAs expressed in bovine macrophages in response to infection with MAP. As expected, pathway analysis revealed the highest enrichment of identified coding genes in general immune related functions such as TNF signalling, NF-κB signalling and TLR signalling. The most upregulated mRNA (ranked by L2FC) was C-C motif chemokine ligand 8 (*CCL8*, Table 1), also known as monocyte chemoattractant protein 2 (*MCP2*) – a pro-inflammatory chemokine which is primarily expressed in monocytes/macrophages e.g. after Interferon (INF) stimulation from T cells or upon recognition of antigens. *CCL8* was demonstrated to be highly expressed in pleural effusions in *M. tuberculosis* infected patients as well as in *M. bovis* BCG and *M. tuberculosis* infected human and mouse macrophages^29^ and is a promising biomarker for detection of tuberculosis^30^. *CCL8* was also among the most upregulated genes in macrophages from red deer and in bovine ileal tissue infected with MAP^31,32^. Furthermore, *CCL8* has been linked to granuloma formation^33^ – a key event in paratuberculosis pathogenesis. In our study, further chemotactic molecules such as *CXCL2*, *CSF2*, *CSF3* and pro-inflammatory cytokines such as *TNF* and *IL-1β* were among the top 20 upregulated coding genes reinforcing that chemokine and cytokine signalling is a central event in early mycobacterial infection *in vitro*^34^.

The data suggests a MAP driven immune response favouring the establishment of infection, as a strong induction of anti-inflammatory mediators such as *IL-10*, *IL-27* and Suppressor of Cytokine Signalling (*SOCS*) 1 and 3 was observed (Supplementary Data 3). These factors have been previously identified as potential key players in the pathogenesis of MAP^6^. As an example, *IL-10* inhibits production of pro-inflammatory cytokines and decreases antigen presentation by macrophages. Mycobacteria-mediated upregulation of *IL-10* is a well-known virulence mechanism to regulate antimicrobial activity resulting in enhanced pathogen survival^35^. Further confirming previous findings, we found a decreased expression of several *RAB* (Ras-related in brain) family members, such as RAB3A, RAB42 or RAB11 family-interacting protein 5 (Supplementary Data 3). These proteins are mainly involved in vesicular trafficking and the coordination of endosomal transport^36^. *RAB3A* was the most downregulated gene in bovine macrophages 2 h after infection with MAP, likely representing a strategy of MAP to inhibit phagosomal maturation to promote its own intracellular survival^37^.

Interestingly, RNA analysis in our study reflects that MAP seems to have a striking impact on INF induced signalling. INFs are cytokines that are widely produced and are involved in the inflammatory response towards several infectious agents and surveillance of malignant cells^38^. We found that several Type I INF response genes were DE by MAP infection, including *ISG20*, *CMPK2* and *IFIT 1* and *2*^39^ (which were also among the top 20 upregulated mRNAs), supporting the relevance of Type I INF signalling in the MAP-macrophage interplay.

In the present study, we identified several genes that have not yet been clearly linked to mycobacterial infections (e.g. leukaemia inhibitory factor, interleukin 6 family cytokine (*LIF*), the schlafen family member 11 (*SLFN11*) or Growth arrest specific 1 (*GAS1*)). *LIF* was among the top five upregulated transcripts. However, the role of *LIF* in bacterial infection is not clear. It was shown to stimulate the production of inflammatory cytokines *IL-1β*, *IL-6*, and *IL-8*^40^. In case of mycobacteriaI infection, it was induced in macrophages infected with *M. smegmatis* but not with pathogenic *M. tuberculosis* or *M. avium*. The authors concluded that it plays a role in clearing non-pathogenic mycobacteria^41^. To our knowledge, it was yet not associated with Paratuberculosis. Another example is *SLFN11*, which has been described as an inhibitor of HIV-1 by interfering with viral protein synthesis^42^. Primary immune cells and other tissue express human *SLFN11* in response to INF Type I signalling^43^. *SLFN11* and other members of the schlafen family have also been shown to be induced by TLR ligands^43^ or bacterial infection pointing to a role in immune processes. However, their role in bacterial infection is not clear. The most downregulated DE coding gene in bovine macrophages in response to MAP was *GAS1*, which plays a role in growth suppression and is a putative tumour suppressor gene^44^. Overexpression induces apoptosis in tumour cells^44^ suggesting a role in the cell cycle or apoptosis. However, it has not been associated to infectious diseases.

### Analysis of lncRNAs point to regulatory functions in the immune response towards MAP infection

In the present study, we were interested in the expression of lncRNAs since they were reported to play a fundamental role in the regulation of the immune response towards several bacterial pathogens^13^ and are known to be influenced by mycobacterial infection^12^. The identification and functional annotation of lncRNAs in cattle is scarce^16^, therefore, one of the main challenges of the study was to identify lncRNAs and infer their biological role. For this, we implemented a computational pipeline based on earlier studies^17–21^ that allowed nominating candidate lncRNAs. As described in these studies, we utilized Tophat2/Cufflinks for the initial alignment and assembly steps. This allowed us to obtain comparable results, especially with respect to previous studies on cattle^28,45^. Moreover, we obtained very good alignment statistics (>90% concordant alignment for all samples, Table S1) with Tophat2, which in comparison to its succeeding software^46^ with faster search algorithm has been shown to provide similar accuracy^46,47^. Additionally, many layers of different information from each step in the pipeline synergistically aided in the identification of potentially functional lncRNA transcripts. As recently shown, ‘the choice of differential expression analysis approach exhibits the strongest impact on results’, with more modest influences from the read aligner and expression modeller^48,49^, we utilized DESeq2 that has been shown to perform robustly in comparison to other existing differential expression tools^50^. Thus, in conjunction with the chosen filtering parameters, coding potential assessment and bioinformatics analysis, we generated a reliable dataset in our study. Nonetheless, we strongly suggest testing the role newer tools, such as pseudoaligners, in future studies with respect to the identification and discovery of unannotated and novel lncRNAs.

Following stringent filtering criteria based on the genomic features comprising exon number, length, expression levels and coding potential, we obtained 397 candidate lncRNAs of which 225 were novel. The genomic features of our candidate lncRNAs were in concordance with earlier observations in cattle and other species^17–21,28,51^, which further establishes the validity of our pipeline. For instance, the candidate lncRNAs possessed lower expression levels (Fig. 2c), fewer exons (Fig. 2a) and the length was typically shorter (Fig. 2b) relative to mRNA, likely attributed to the fewer number of exons in the transcript. The identified lncRNAs exhibited lower average conservation across the transcripts in comparison to protein-coding mRNA, which may provide them the ability to undergo rapid functional diversification. For instance, the validated non-coding RNA gene locus *XLOC_000633* had a very low average conservation score of 0.0331. However, in agreement to earlier studies^20,52^, we observed local regions displaying reasonable conservation (Supplementary Data 2) relative to the rest of the transcript, showing that lncRNAs display more positional conservation than sequence conservation.

We observed an overlap between few predicted lncRNA candidates and the previously published non-coding RNAs from five different sources for cattle. Although, the previously published non-coding RNAs were identified in different types of tissue, it is likely that some of the candidate lncRNAs share a common functionality and expression with their overlapping counterpart, pointing towards the fact that the expression of some of these lncRNAs may not be cell-specific. However, to the best of our knowledge, inadequate information existed regarding the functionality of these previously published non-coding RNAs. Therefore, a fundamental question raised following the identification of novel lncRNAs is regarding their relevance in biological function. To address this question, we utilized the fact that lncRNA fine-tune the expression of neighbouring genes^53^. Interestingly, on comparing the distribution of lncRNAs in relation to mRNAs across the genome (as described earlier in the results section), we observed that several DE lncRNAs were present on chromosomes 18 and 19, a pattern analogous to DE mRNA. Interestingly, chromosomes 18 and 19 harbour some of the protein-coding genes associated with signalling pathways for innate immune response. For instance, DE genes on chromosomes 18 and 19 are involved with TNF signalling pathway (*CCL5*, *CCL2*, *MAP2K3*, *MAP2K4*, *MAP3K14*, *MLKL*, *PIK3R5* and *SOCS3*), chemokine signalling pathway (*CCL5*, *NFKBIB*, *ARRB2*, *CCL2*, *CCL4*, *CCL8*, *PIK3R5*), NOD-like receptor signalling pathway (*CCL5*, *NFKBIB*, *NLRP1*, *CCL2*) and cytokine-cytokine receptor interaction (*CCL5*, *CCL2*, *CCL4*, *CCL8*, *CSF3*). To investigate the functional aspect in more detail, we analysed the neighbouring genes of the lncRNAs. Interestingly, we observed differential expression of corresponding mRNA neighbours for 20 DE lncRNAs. The co-expression analysis indicated that these neighbouring lncRNA-mRNA pairs showed changes in expression along the same direction (Fig. 4). Notably, the distance between DE lncRNA-mRNA gene pairs was much shorter than the commonly observed distance between lncRNA-mRNA pairs (as described in the results section), which may support the hypothesis that these lncRNA participated in cross-regulation. Enrichment analysis clearly showed that neighbouring DE mRNAs were involved in pathways related to immune response (Supplementary Fig. S6). For example, this included protein coding genes that play a role in NF-κB2 signalling such as TNFAIP3 interacting protein 3 (*TNIP3*), TNF alpha induced protein 3 (*TNFAIP3*) and *NF-κB2*.

TNIP3 and TNFAIP3 negatively influence NF-κB signalling. *TNFAIP3* is induced by TNF and limits inflammation by terminating TNF induced NF-κB response^54^. The murine lncRNA *lincRNA*-*TNFAIP3*, located proximal to the *TNFAIP3* gene, was shown to be an early-primary response gene controlled by NF-κB signalling in murine macrophages^55^. The authors demonstrated that lincRNA-TNFAIP3 seems to assemble a NF-κB/HMGB1/lincRNA-TNFAIP3-complex in response to lipopolysaccharides which leads to epigenetic chromatin remodelling and transactivation of inflammatory genes. It was recently shown that a lncRNA adjacent to *TNFAIP3* was among eight lncRNAs that were exclusively expressed in *Campylobacter* *concisus* infected THP-1 macrophages accompanied by co-expression of *TNFAIP3*^56^. Strikingly, in our study the TNFAIP3 interacting protein (*TNIP3*) was also differentially regulated as well as the adjacent lncRNA further suggesting that MAP-induced lncRNAs influence macrophage response towards infection. Regulation of *TNFAIP3* during mycobacterial infection has been shown for miRNAs leading to limitation of NF-κB inflammatory signalling which favours mycobacterial survival^57^. Thus, regulation of NF-κB signalling by *TNFAIP3* may be orchestrated by multiple classes of non-coding RNAs including lncRNAs.

Interestingly, in our study, two protein coding genes (*IFI44F* and *MX2*) that have been implicated in the cellular response to type I interferons^58,59^ as well as their neighbouring lncRNAs were significantly upregulated in response to infection suggesting a supportive role of lncRNAs in type I INF signalling during MAP infection. In accordance with that assumption, it has recently been shown that type I INFs regulate the expression of lncRNAs and vice versa^60^. However, to which extent the identified lncRNAs in our study contribute to the regulation of INF signalling during MAP infection has to be analysed in more depth in the future.

Among the possible mode of actions of lncRNAs, their regulatory role may be either inhibiting or stimulating gene expression. In our study, several lncRNAs were highly regulated by the infection, however, their functional role remains to be investigated and validated in terms of regulatory mechanisms. The potential of lncRNAs to serve as a biomarker has previously been demonstrated in *M. tuberculosis* infection^12^ and may also be a promising approach in case of Paratuberculosis.

In conclusion, we have shown that MAP induces an expression profile of genes related to immune response and bactericidal defence mechanisms in bovine macrophages. Our data support existing knowledge about the ability of MAP to subvert the host immune defence. However, we also identified DE genes, which have not yet been associated with Paratuberculosis infections, opening new insight into the pathogenic mechanisms employed by MAP or yet unknown defence strategies used by the host cell. To our knowledge, this is the first study that provides novel insights into the role of lncRNAs and their interplay with mRNAs during MAP infection in cattle.

Whether identified lncRNAs are specific to MAP infection and may serve as potential biomarkers to detect infected animals has to be the matter of future investigations.

## Materials and Methods

### Isolation and Cultivation of Primary Bovine Macrophages

Whole blood of three 15 month old Holstein Friesian cows was obtained from the jugular vein under sterile conditions and kept in Na-citrate to prevent coagulation. Animals were tested negative for MAP infection by ELISA and fecal culture (Laboklin, Bad Kissingen, Germany). Blood mononuclear cells were isolated by Ficoll-Paque Plus (GE Healthcare, Sigma-Aldrich, Taufkirchen, Germany) centrifugation, three washing steps with PBS and subsequent attachment to cell-culture plate surface. Cells were cultivated at 37 °C and 5% CO_2_ for 6 days in six well plates in 1.5 ml IMDM medium containing 10% FCS, 10 μg/ml gentamycin (Biochrom, Berlin, Germany) and 30 ng/ml M-CSF (PAN Biotech, Aidenbach, Germany) and 1 x Penicillin-Streptomycin-Amphotericin B (Sigma-Aldrich, Taufkirchen, Germany). The media was changed one to two times during cultivation.

Flow cytometry was performed to analyse CD14 expression, a cell surface marker for monocytes and macrophages, to reveal the purity of the isolated macrophage population. For that purpose, part of the isolated cells were stained with a CD14 antibody as well as a secondary fluorochrome-labelled antibody and analysed with a FACSCalibur flowcytometer (Becton Dickinson GmbH, Heidelberg, Germany) as described previously^61^. The fluorochrome-stained cells were gated and analysed for CD14 antibody staining. If FACS analysis proved that the analysed cell population contained around 90% CD14 + cells (Supplementary Fig. S8), cells were washed with PBS and fresh IMDM media was supplied containing 10% FCS. For quantification, two wells were counted by detaching the cells with Accutase (PAA, Pasching, Austria) and cell number was determined by staining the cells with trypan blue and counting in a Newbauer-Chamber. Subsequently, cells were infected as described below.

### Bacterial strain and culture condition

MAP (ATCC 19698) was cultured at 37 °C on Herrold’s Egg yolk slants (BD Life Sciences, Heidelberg, Germany) or Agar plates (1 l containing 900 ml Aqua dest, 4.7 g Middlebrook-Bouillon 7H9 (BD Life Sciences, Heidelberg, Germany), 2 ml Glycerin (Roth, Karlsruhe, Germany), 0.5 g Tween 80 (Roth, Karlsruhe, Germany), 16 g Agar-Agar (Roth, Karlsruhe, Germany), 100 ml Middlebrook OADC-Supplement (BD Life Sciences, Heidelberg, Germany) and 2 ml Mycobactin J (ID Vet, Grabels, France). Cultures were transferred from slants or plates to Middlebrook-Bouillon (1 l containing 900 ml Aqua dest, 4.7 g Middlebrook-Bouillon 7H9, 2 ml Glycerin, 0.5 g Tween 80, 100 ml Middlebrook OADC-Supplement and 2 ml Mycobactin J) and kept at 37 °C until the culture reached OD_600_ = 1. Bacteria were frozen in PBS containing 10% Glycerol until used for infection experiments. For quantification of bacteria, the number of colony forming units was determined by plating serial dilutions on 7H9 agar plates which were incubated at 37 °C until cultures were visible.

### Infection experiments

Approximately 2 × 10^7^ bacterial cells were inoculated on 2 × 10^5^ primary bovine macrophages (multiplicity of infection [MOI] = 100) and incubated at 37 °C and 5% CO_2_. Non-infected cells served as a negative control. After 2 h of infection, cells were washed three times with PBS and incubated in fresh media containing 10% FCS for another 4 h to remove remaining extracellular bacteria. Samples were taken 6 h after infection. For RNA extraction, cells were washed three times with PBS, lysed with RNA lysis buffer (mirVANA, Life Technologies, Darmstadt, Germany) and total RNA was isolated according to the manufacturer’s instruction.

### Quality control of isolated RNA

Quantity and quality of RNA were first determined by measuring absorbance at 260 and 280 nm with a Nano Drop 1000 spectrophotometer according to the manufacturer’s instructions (Thermo Fisher Scientific, Germany). Samples were further analysed for their RNA integrity with an Agilent 2100 BioAnalyzer and RNA 6000 Nano Kits (Agilent, Waldbronn, Germany) according to the manufacturer’s protocol. RNA with integrity value (RIN) of ≥ 8.8 was used for further investigation.

### RNA library preparation and sequencing

Sequencing of total RNA was performed at the Institute of Clinical Molecular Biology at Christian-Albrechts-University Kiel for six samples, where both infected and control samples consisted of three biological replicates. mRNA and lncRNA expression were derived from same total RNA sequencing. Input RNA quantity was assessed using a Qubit 2.0 Fluorometer and the Qubit RNA BR Assay Kit according to manufacturer’s instructions. Sequencing libraries were constructed using the Illumina TruSeq stranded total RNA kit according to manufacturer’s instructions with an input of 500 ng total RNA per sample. This kit includes ribosomal RNA removal by Ribozero treatment. The quality of final libraries was checked on a TapeStation2200 using the D1000 Screen Tape. Libraries were sequenced on an Illumina HiSeq4000 (Illumina) with 2 × 75 bp paired end reads and six samples per lane.

### Data processing, mapping and transcriptome assembly

For the initial steps of the analysis, mainly involving sequence alignment and assembly, we utilized the main cloud-computing server of Galaxy (usegalaxy.org), which provides an open source, web-based platform for the next generation sequencing specific analysis. Prior to mapping and assembly, the raw RNA-Seq fastq files were transformed (FASTQ Groomer, Galaxy version 1.0.4) to a format compatible with Galaxy and assessed for quality using FastQC (Galaxy version 0.68). The paired-end reads from each sample were aligned to the cow genome (bosTau8 June 2014) using the spliced-read aligner Tophat2^62^ (Galaxy version 2.1.0). An *ab initio* transcriptome assembly was performed with Cufflinks^63^ (Galaxy version 2.2.1) using uniquely mapped and properly paired reads to identify annotated and novel transcripts in the data. Finally, using Cuffmerge^63^ (Galaxy version 2.2.1.0) we created a single assembly GTF file from the individual Cufflinks assemblies. In order to maximize the overall assembly quality with Cuffmerge, we provided the cow genome and annotation files (Ensemble UMD3.1, https://support.illumina.com/sequencing/sequencing_software/igenome.html) as inputs.

### Filtering of transcripts

To select potential long non-coding RNAs from the transcripts generated by Cufflinks, firstly, we retained transcripts with no known annotation (novel intergenic: class code u and novel antisense: class code x). In the next step, we discarded transcripts with a length less than 200 bp. It has been shown that many lncRNAs consist of single-exon transcripts^18,28,64^. However, often single-exon transcripts are discarded as they can potentially contribute to background noise resulting from erroneous transcript assembly, genomic contamination in the RNA-Seq library or experimental artifacts. An earlier study in humans^20^ applied a stringent size threshold of 1 kb for selecting single exon transcripts to overcome the above-mentioned problems. We applied a size threshold of 500 bp (similar to that reported in a recent study by Bush *et al*.^65^) to single-exon transcripts, considering the above-mentioned limitations and the fact that the average length of a single-exon transcripts in cattle is reported to be 286 bp^64^. We estimated number of fragments in all paired-end libraries using featureCounts^66^ (version 1.5.2), and normalized the expression in terms of fragments per kilobase of transcript per million mapped reads (FPKM). Finally, to select expressed versus background transcripts, we discarded any transcript having an abundance of less than 1.5 FPKM in all six samples based on earlier studies^17,20,67^. However, we strongly recommend investigating the distribution of transcript abundance for selecting a reasonable cut-off.

### Characterization of the coding potential

We used coding potential calculator^23^ (CPC; version cpc-0.9-r2), Coding-Potential Assessment Tool^24^ (CPAT; version 1.2.2) and hmmscan algorithm (version HMMER 3.1b2, http://hmmer.org/) to examine the protein coding ability of the shortlisted transcripts. CPC and CPAT utilize sequence features to distinguish coding from non-coding RNAs, therefore, we extracted DNA sequences of the transcripts using Cufflinks’ gffread^63^. To classify a transcript as non-coding, we chose a stringent threshold of −0.5 for CPC to reduce possible false positive classification^18^. Next, we used CPAT to assess the protein coding potential of candidate transcripts. In order to choose the cut-off threshold for cattle, we prepared a training dataset consisting of 10,000 known ‘coding sequences’ and 10,000 ‘non-coding sequences’ (UMD3.1; ftp://ftp.ensembl.org/pub/release-91/fasta/bos_taurus/), similar to Billerey *et al*.^19^. As only 3,801 sequences were available for the non-coding RNAs, we used 6,199 intron sequences for creating a balanced training dataset. Both the coding and intronic sequences were selected randomly. We performed 10-fold cross-validation to obtain the optimal cut-off value from the two-graph ROC curve. In comparison to other studies, we also arrived at a similar^19^ cut-off value of 0.34 (Supplementary Fig. S9). Lastly, we used the hmmscan algorithm to scan the Pfam protein domains in the candidate lncRNAs. The sequences were translated into the three open reading frames using EMBOSS transeq^68^ (EMBOSS: 6.5.7.0) and queried against the Pfam protein profile database^22^ (version Pfam31.0) using hmmscan. Candidate lncRNAs with a match to a domain in a known protein sequence were discarded. Finally, we chose only those transcripts as non-coding RNAs that did not possess coding potential with respect to any of the three above-mentioned tools.

### Conservation analysis

We downloaded the available PhastCons scores^25^ from UCSC (http://hgdownload.soe.ucsc.edu/goldenPath/bosTau4/phastCons5way/) for multiple alignments of four vertebrate genomes (dog, human, mouse and platypus) to the cow genome. For extracting the conservation scores for our genomic region of interest, we converted the file to BED format using bedops^69^ (version 2.4.26). Since the PhastCons scores are only available for the bosTau4 assembly, we used the ‘liftover’ functionality of UCSC to convert the bosTau8 assembly based genome coordinates of our candidate lncRNAs to bosTau4. Finally, we computed an average conservation score from the base-by-base conservation scores for our candidate lncRNA transcripts.

### Nearest neighbour analysis to predict putative target genes

Using bedtools closest^69^ (version 2.26.0), we searched for the nearest cis/trans and upstream/downstream non-overlapping neighbours of the lncRNAs with respect to known annotated genes (Ensemble UMD3.1). Thereafter, we deduced the biological functionality of our candidate lncRNAs by investigating the properties (such as biological role and level of expression) of its closest identified gene.

### Comparison to known lncRNAs

We compared the overlap of our lncRNAs to previously published lncRNAs^18,19,28^ and lncRNAs in the NONCODE (NONCODE2016_bosTau6) and ALDB (ALDB.cow.lincRNAs.v1.0) databases for cattle. Using bedtools intersect^69^ (version 2.26.0), we reported the ‘degree of overlap’ as the number of nucleotides shared between the two overlapping features on the same strand. Since the annotation BED file in the NONCODE database is based on the bosTau6 assembly, we used the ‘liftover’ functionality of UCSC to covert the BED file from the bosTau6 to the bosTau8 assembly.

### Differential expression, gene set enrichment and pathway analysis

Differential expression analysis was performed using the count-based method DESeq2^70^ (version 1.16.1) that utilizes a negative binomial distribution to model the count. A gene was considered significantly DE if p.BH <0.05.

To infer the biological function of the gene expression data, we performed gene set enrichment analysis for the DE genes using Functional Annotation Tool in DAVID^27^ (version 6.8). Using the DE protein-coding gene list as an input to DAVID and *Bos tau* as background, we obtained annotation terms pertinent to MAP infection. DAVID provided EASE score (a modified Fisher Exact p-value) as well as p.BH signifying enriched biological annotations. An EASE score <0.05 was used as a cut-off.

### Validation of selected lncRNAs using RT-qPCR

cDNA was synthesized from two RNA samples (the same that were used for RNA-Seq) of infected as well as non-infected macrophages with the Maxima First Strand cDNA synthesis kit (Thermo Scientific) as described in the manufacturer’s protocol. Pooled cDNA was taken as a template for testing the primers listed in Supplementary Table S5 via gradient RT-qPCR and gel electrophoresis of PCR products. Amplicons showing the expected size were isolated and sequenced to ensure specificity. Expression analysis was performed by means of SYBR Green detection chemistry using the SensiMix SYBR Hi-ROX Kit (Bioline GmbH) as described before^71^. A PikoReal Cycler was used instead of a Step one plus. Expression of housekeeping genes *GAPDH* and *SDHA* was analysed in parallel to normalize the data. The ΔΔCT method^72^ was used to calculate the relative fold difference of RNA expression levels compared to the negative control.

### Ethics

The study was approved by the local animal welfare committee of the regional authorities at the federal state Brandenburg, Germany (No. 2347-A-3-1-2016) and carried out in accordance with the approved guidelines and regulations.

## Supplementary information

Supplementary Information

Supplementary Dataset 1a

Supplementary Dataset 1b

Supplementary Dataset 2

Supplementary Dataset 3

Supplementary Dataset 4

Supplementary Dataset 5

Supplementary Dataset 6

## Data Availability

All data generated or analysed during this study are included in this published article (and its Supplementary Information files). The raw RNA-Seq fastq data discussed in this publication have been deposited in NCBI’s Gene Expression Omnibus^73^ and are accessible through GEO Series accession number GSE122933 (https://www.ncbi.nlm.nih.gov/geo/query/acc.cgi?acc = GSE122933).
