# Supplementary material for: Analysis of long non-coding RNA and mRNA expression in bovine macrophages brings up novel aspects of Mycobacterium avium subspecies paratuberculosis infections
Source: Sci Rep. 2019 Feb 7;9:1571. doi: 10.1038/s41598-018-38141-x (PMC6367368; doi:10.1038/s41598-018-38141-x)
Supplement: Supplementary file 1 — Supplementary Information [file 41598_2018_38141_MOESM1_ESM.pdf]

Analysis of long non-coding RNA and mRNA expression in bovine macrophages brings up novel aspects of *Mycobacterium avium* subspecies *paratuberculosis* infections

Pooja Gupta[1,6,*], Sarah Peter[2], Markus Jung[2], Astrid Lewin[3], Georg Hemmrich-Stanisak[4], Andre Franke[4], Max von Kleist[1], Christof Schütte[1,6], Ralf Einspanier[5], Soroush Sharbati[5], Jennifer zur Bruegge[5]

[1] Department of Mathematics and Informatics, Freie Universität Berlin, Germany

[2] Institute for the Reproduction of Farm Animals Schönow Inc., Bernau, Germany

[3] Robert Koch-Institute, Department Infectious Diseases, Berlin, Germany

[4] Institute of Clinical Molecular Biology, Christian-Albrechts-University Kiel, Germany

[5] Institute of Veterinary Biochemistry, Department of Veterinary Medicine, Freie Universität Berlin, Germany

[6] Department of Mathematics for Life and Materials Sciences, Zuse Institute Berlin, Germany

***Correspondence:** gupta@math.fu-berlin.de

Supplementary Tables

Supplementary Table S1. Tophat alignment statistics

Sample ID	Treatment	Input read pairs	Aligned read pairs	Concordant pair alignment rate
E035	Infected	52826352	48540359	90.8%
E036	Control	59397740	54341676	90.3%
E037	Infected	56967955	52463220	90.7%
E038	Control	50952922	46761080	90.7%
E039	Infected	61426777	56290035	90.4%
E040	Control	57841437	52839794	90.0%

Supplementary Table S2. Number of transcripts at each step of filtering.

Filtering step	Number of Non-Coding Candidates
Unannotated transcripts (class code u/x)	14295
FPKM > 1.5	1184
Length > 200 and for single exon > 500	931
No match with the Pfam database	490
Protein coding potential (CPC + CPAT)	397

Supplementary Table S3. Distribution of candidate lncRNA across the cow genome.

Chromosome	Chromosome length (bp)	Number of transcripts	Per 10 MB	Percentage transcripts
1	158337067	8	0.51	1.04
2	137060424	12	0.88	1.80
3	121430405	29	2.39	4.90
4	120829699	11	0.91	1.87
5	121191424	18	1.49	3.05
6	119458736	16	1.34	2.75
7	112638659	18	1.60	3.28
8	113384836	10	0.88	1.81
9	105708250	4	0.38	0.78
10	104305016	21	2.01	4.13
11	107310763	17	1.58	3.25
12	91163125	9	0.99	2.02
13	84240350	6	0.71	1.46
14	84648390	12	1.42	2.91
15	85296676	21	2.46	5.05
16	81724687	20	2.45	5.02
17	75158596	13	1.73	3.55
18	66004023	23	3.48	7.15
19	64057457	17	2.65	5.44
20	72042655	12	1.67	3.42
21	71599096	13	1.82	3.72
22	61435874	13	2.12	4.34
23	52530062	6	1.14	2.34
24	62714930	8	1.28	2.62
25	42904170	11	2.56	5.26
26	51681464	5	0.97	1.98
27	45407902	10	2.20	4.52
28	46312546	4	0.86	1.77
29	51505224	18	3.49	7.17
X	148823899	12	0.81	1.65

Supplementary Table S4. Number of candidate lncRNA having an overlap with previously published lncRNAs.

Source	Strand information	Overlapping number of isotigs
NONCODE2016_bosTau6	Provided	85
ALDB.cow.lincRNAs.v1.0	Provided	63
published_billerey_4closest	Provided	36
published_koufariotis_4closest	Not provided	53
published_weikard_4closest	Not provided	44

Supplementary Table S5. Primer sequences to validate lncRNA expression via RT-qPCR (annealing temperature 60°C).

Gene ID	fw 5‘-3‘	Rev 5‘-3‘
XLOC_000633	TTTACATCCTGCCTTGGGGG	AAATCCAAGACTGCGAGCCA
XLOC_030080	GTTGGTGCCTTGGGTACTGA	AGGAAAGTCAGCCAAGTGGT
XLOC_029370	CTGAGCACGAGGTTCTCTGG	GAGTCTTTGAAGGGTGGGGG

Supplementary Figures

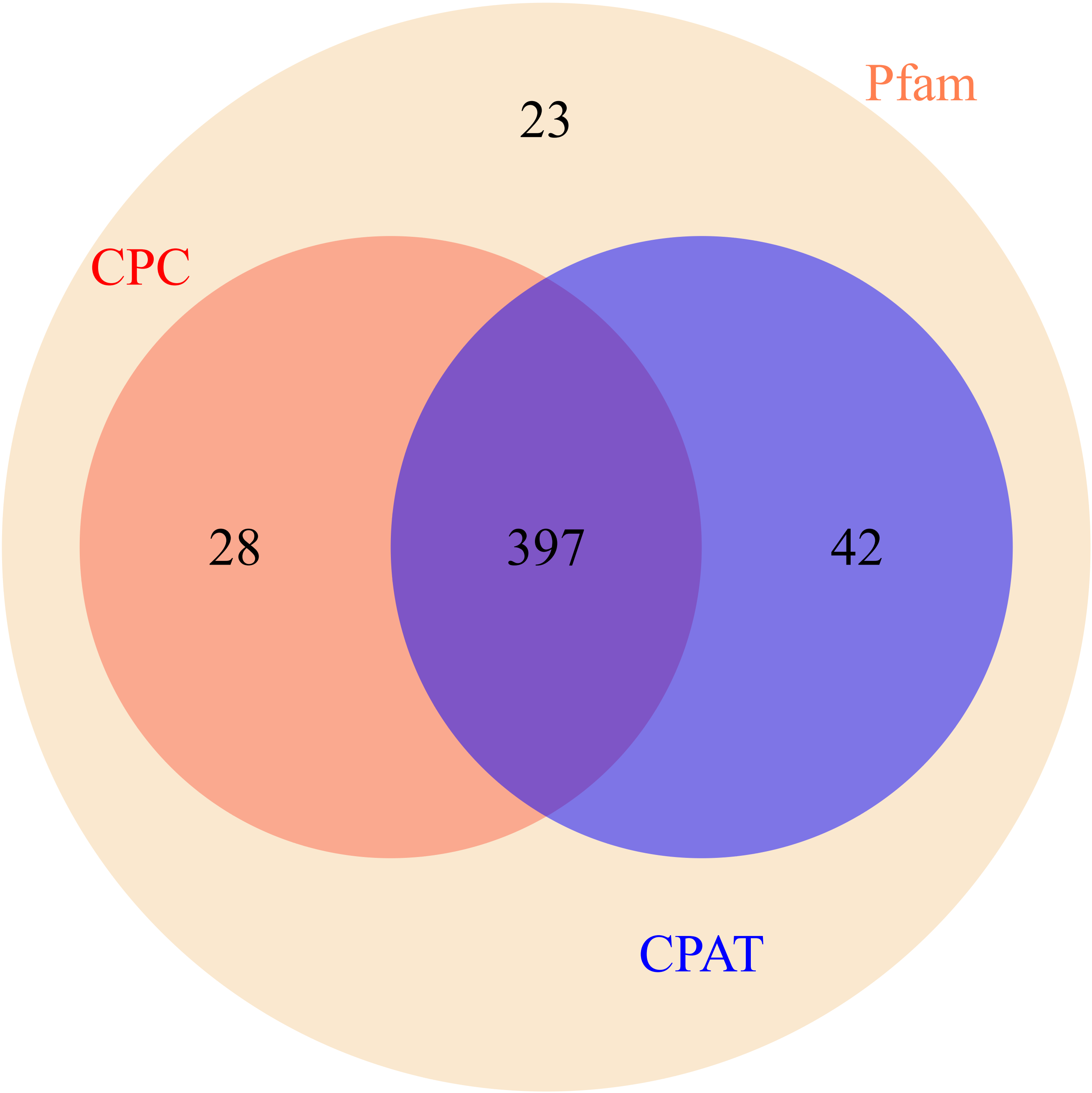

Supplementary Fig. S1. Coding potential analysis. Venn-Diagram showing the number lncRNA selected by CPAT, CPC and hmmscan based search of the Pfam database and an overlap between different methods.

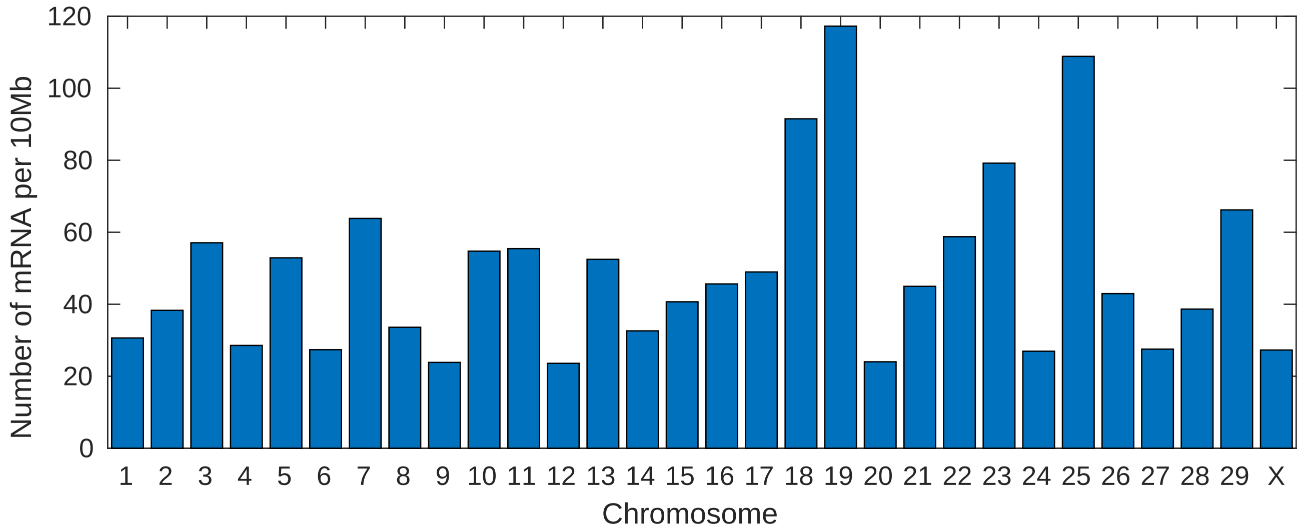

Supplementary Fig. S2. Genomic distribution of mRNA. Bar plot showing the distribution of mRNA expressed in our samples across the cow genome.

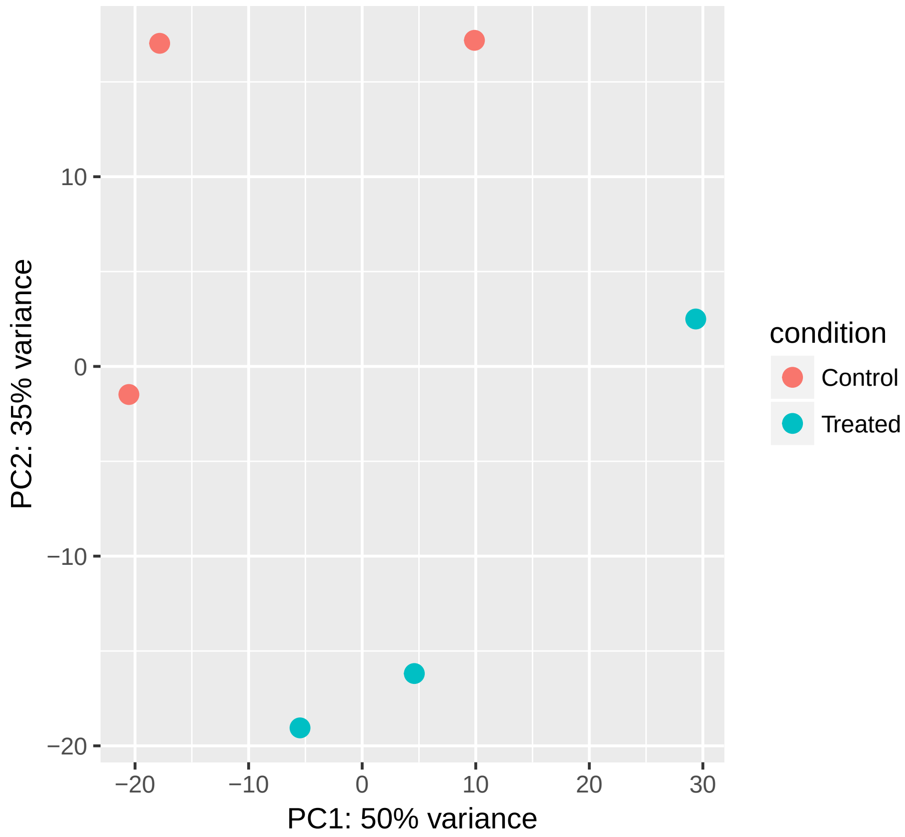

Supplementary Fig. S3. Principal component plot of the samples. Principal component analysis providing an initial assessment of sample distance.

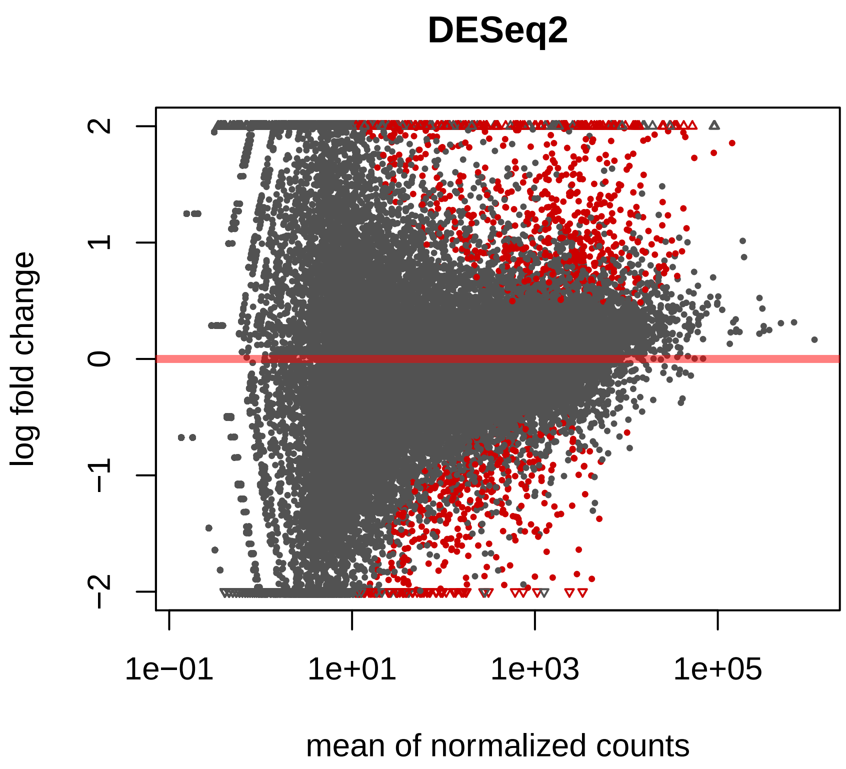

Supplementary Fig. S4. MA-plot. MA-plot showing the log2 fold changes over the mean of normalized counts for all the samples due to infection. Points colored in red indicate that the 'Benjamini-Hochberg corrected p-value' is less than 0.05.

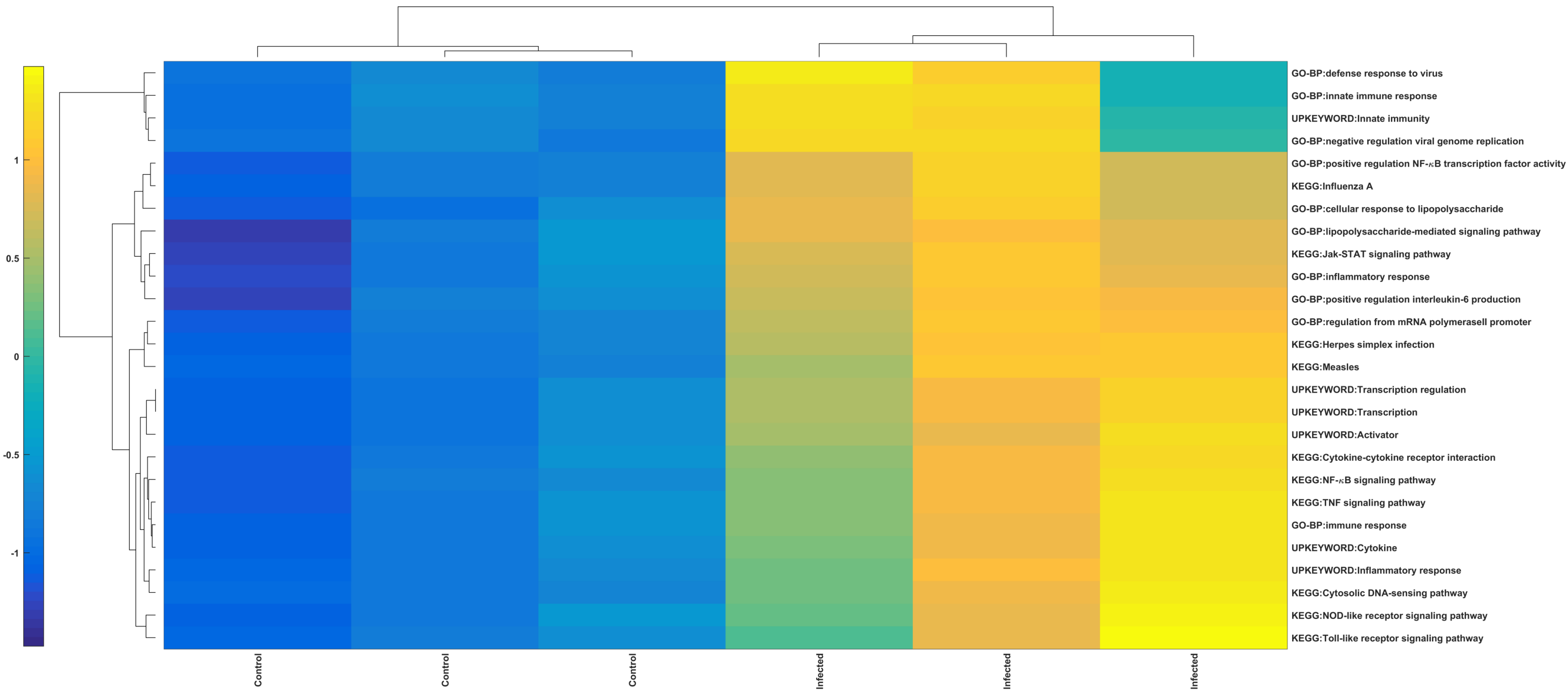

Supplementary Fig. S5. Enrichment analysis for the differentially expressed mRNA. A hierarchical heatmap showing significantly enriched gene-ontology biological processes (GO-BP), KEGG terms and UPKEYWORD ('Benjamini-Hochberg corrected p-value' < 0.05) for the differentially expressed mRNA in control and infected samples. The yellow and blue colors indicate higher and lower expression of genes associated with the significantly enriched terms.

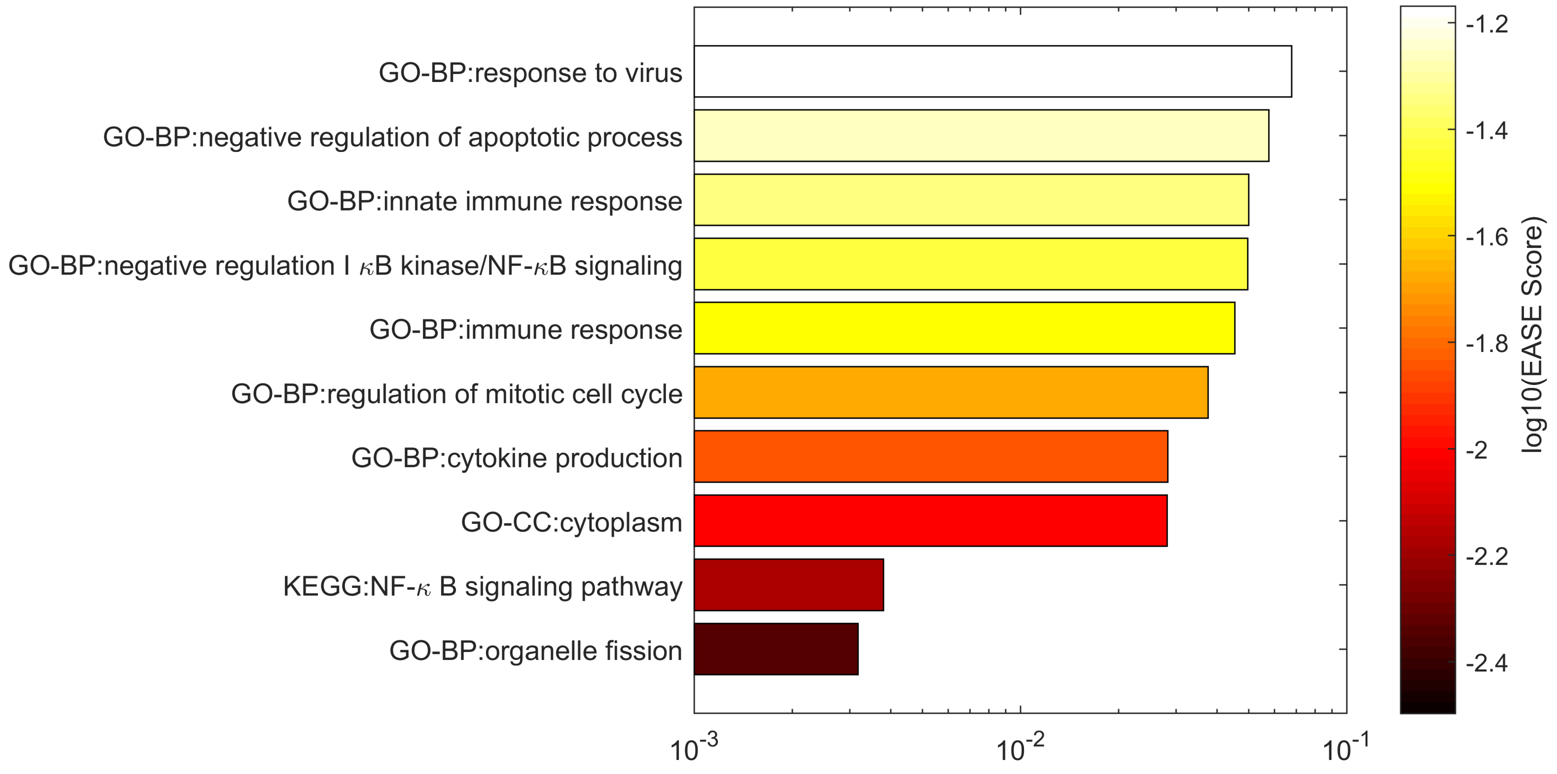

Supplementary Fig. S6. Top enrichment terms for the neighboring mRNA of the differentially expressed lncRNA. An overview of the significantly enriched terms (EASE score < 0.05) for the neighboring mRNA of the differentially expressed lncRNA in the form of a bar plot. The length of the bar plot indicates the EASE score.

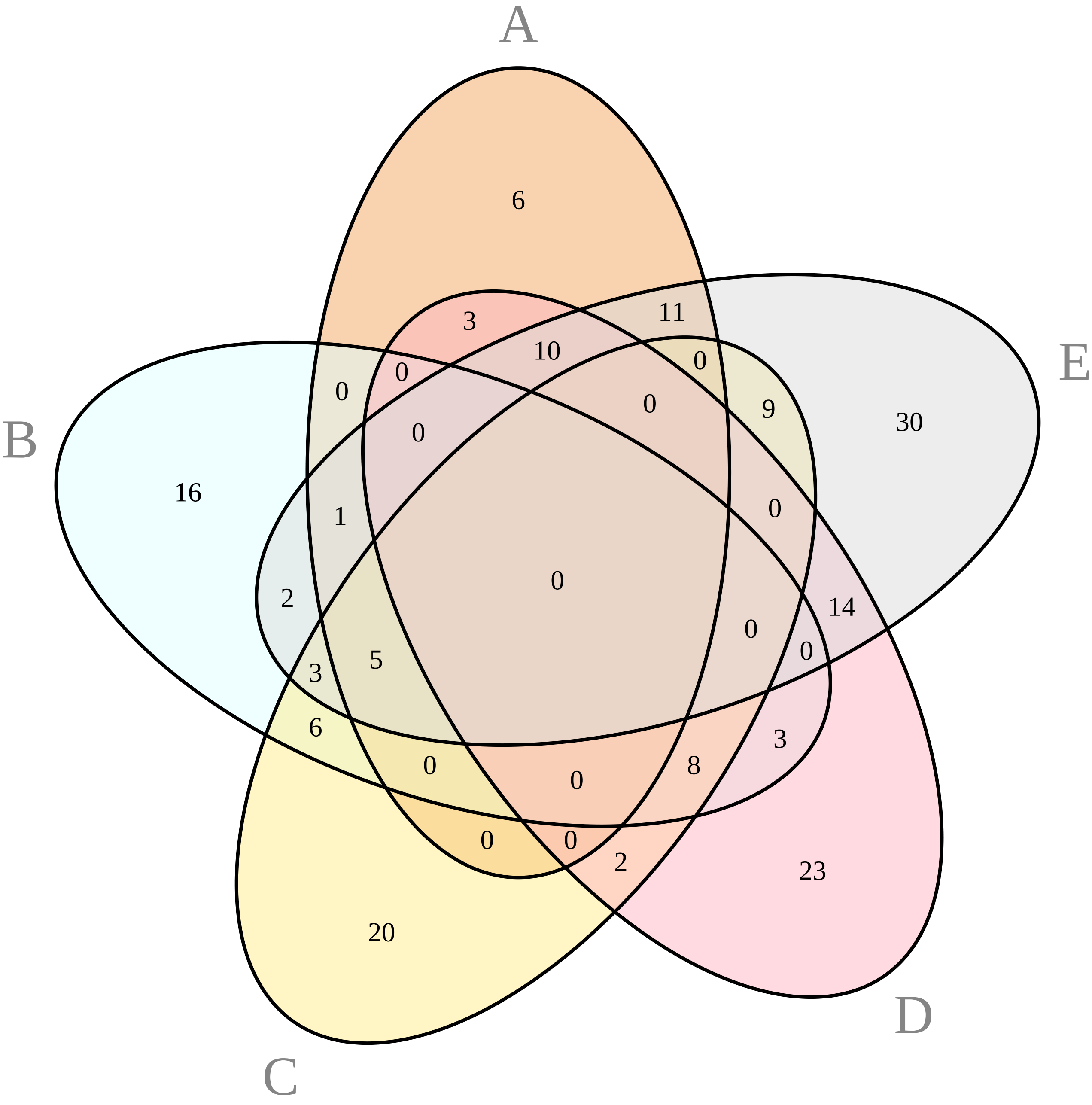

Supplementary Fig. S7. Overlap with previously published lncRNAs. Venn diagram showing the number of candidate lncRNA having an overlap with previously published lncRNAs. A: Billerey et al. 2014; B: Koufariotis et al. 2015; C: Weikard et al. 2013, D: NONCODE database and E: ALDB database.

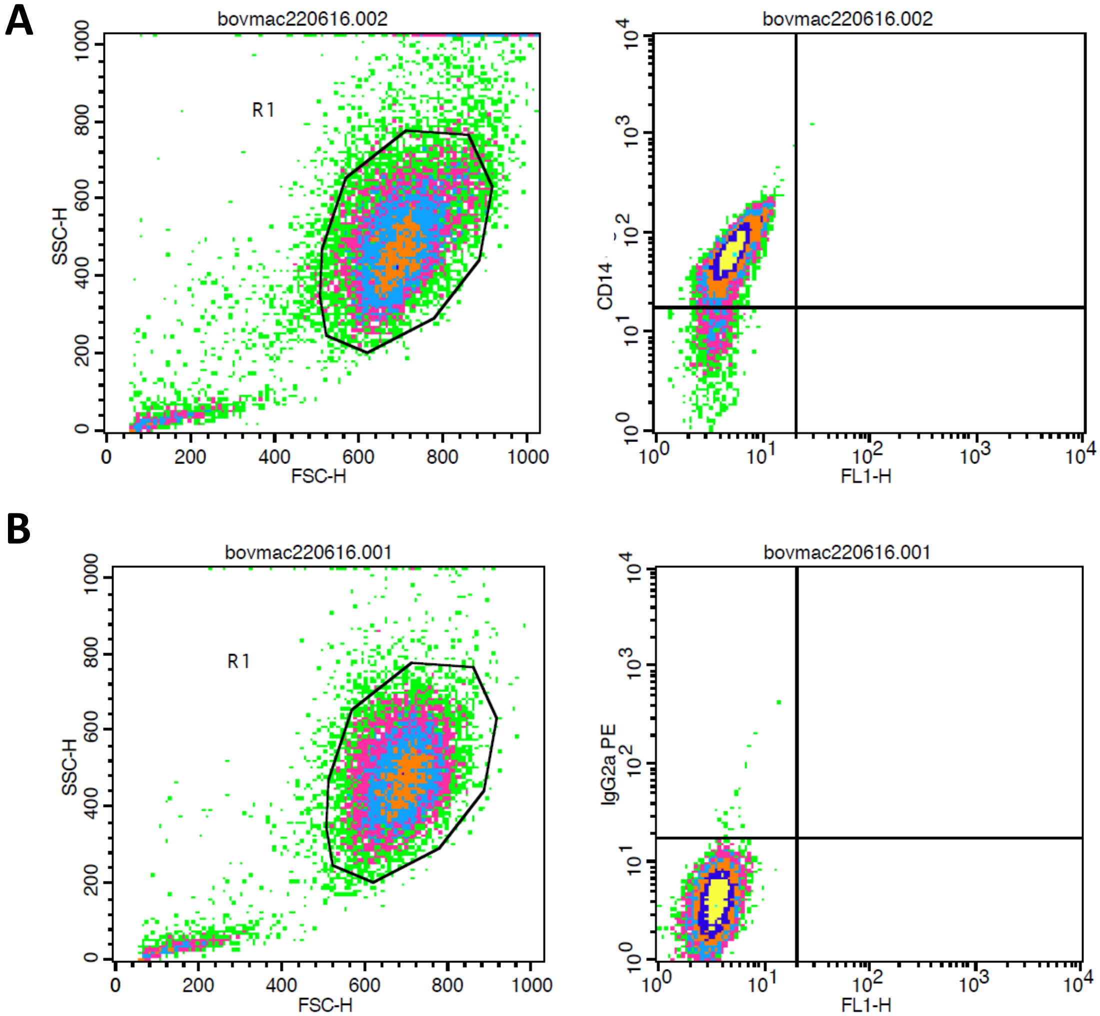

CD14+

Quad	Events	%Gated	%Total
UL	10180	90.88	74.40
UR	1	0.01	0.01
LL	1021	9.11	7.46
LR	0	0.00	0.00

IgG2α control

Quad	Events	%Gated	%Total
UL	25	0.24	0.21
UR	0	0.00	0.00
LL	10502	99.76	88.62
LR	0	0.00	0.00

Supplementary Fig. S8. FACS plots of CD14 positive primary bovine macrophages. (A) FACS plot indicates 90.88% CD14+ of gated cells with (B) 0.24% unspecific binding of the secondary antibody (control, IgG2α). The lower right (LR) quadrant represents early apoptosis, lower left (LL) quadrant represents viable cells, upper right quadrant (UR) represents late apoptosis and upper left quadrant (UL) represents necrosis.

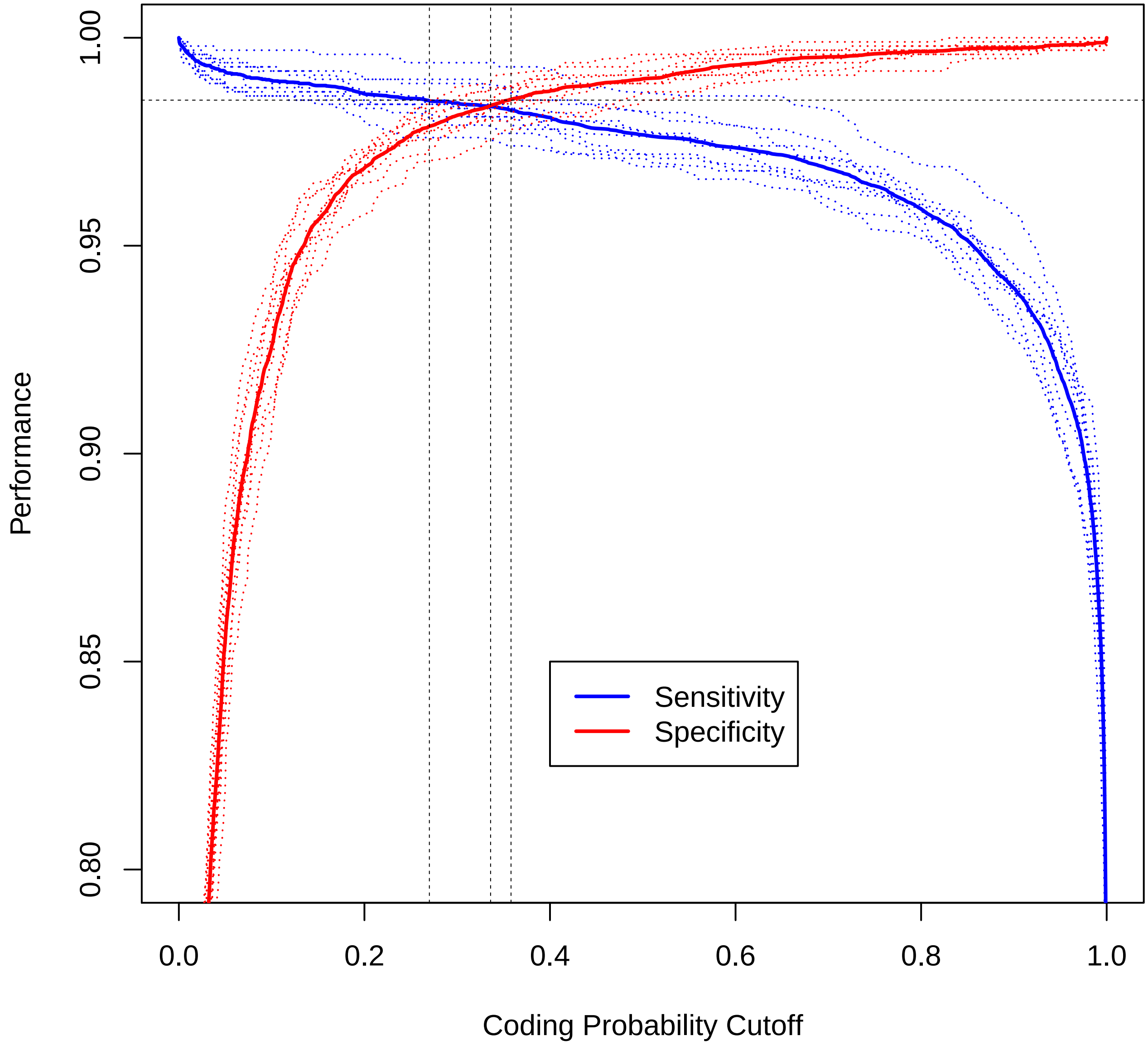

Supplementary Fig. S9. Two-graph ROC curve. An optimumcut-off value of 0.34 was obtained for cow using the two-graph ROC for CPAT.
